# Human perception of spatial frequency varies with stimulus orientation and location in the visual field

**DOI:** 10.1038/s41598-023-44673-8

**Published:** 2023-10-17

**Authors:** Wladimir Kirsch, Wilfried Kunde

**Affiliations:** Department of Psychology, University of Würzburg, Röntgenring 11, 97070 Würzburg, Germany

**Keywords:** Neuroscience, Psychology

## Abstract

Neuroanatomical variations across the visual field of human observers go along with corresponding variations of the perceived coarseness of visual stimuli. Here we show that horizontal gratings are perceived as having lower spatial frequency than vertical gratings when occurring along the horizontal meridian of the visual field, whereas gratings occurring along the vertical meridian show the exact opposite effect. This finding indicates a new peculiarity of processes operating along the cardinal axes of the visual field.

## Introduction

Performance in many visual tasks is inferior for stimuli occurring in the periphery rather than the center^1^ and along the vertical than along the horizontal meridian of the visual field^2^. These differences in visual perception are related to neuroanatomical characteristics of the visual system. While the link between anatomy and performance is not fully understood, increasing size of cortical receptive fields (RF) and decreasing cortical magnification (cortical surface dedicated to a particular region of the visual field) can account for a drop in visual performance with stimulus eccentricity^3–5^. In a similar vein, larger cortical magnification and smaller RFs in the horizontal than in the vertical meridian have been linked to better performance along the horizontal than along the vertical meridian^6–8^. Here we report a psychophysical finding that indicates a yet unknown peculiarity of this link between perception and brain anatomy.

We assessed the perception of spatial frequency (SF), i.e., how coarse or fine-grained stimuli are perceived. It is known that the same physical stimulus can appear more or less fine-grained to human observers. For example, objects appear coarser after adapting to a stimulus of a higher SF^9^, with a higher level of stimulus luminance^10^, with longer presentation duration^11^, and with a decrease of eccentricity^12^.

In the present study, we focused on another striking observation that horizontal gratings are perceived as coarser than vertical gratings of the same frequency^12–14^. This finding has been originally referred to the “vertical-horizontal illusion”—the tendency to see vertical lines as longer than equally long horizontal lines^13^. The core idea was that spatial distances are perceived as smaller along the horizontal than along the vertical meridian indicating that the perceptual space is compressed along the horizontal meridian relative to the vertical meridian. This assumption seems consistent with the asymmetries in visual performance and cortical tissue between horizontal and vertical meridians mentioned above. Notably, in the original experiments, stimuli were usually presented pairwise side by side, i.e., along the horizontal meridian of visual field^12–14^. We wondered whether the reported difference in perception between horizontally and vertically oriented gratings holds true when stimuli are presented along the vertical and thus examined how this effect depends on whether the stimuli vary along the horizontal or vertical meridian of the visual field.

We first confirmed previous observations that horizontal gratings appear coarser than vertical gratings when these gratings occur along the horizontal meridian. Yet, when stimuli were presented along the vertical meridian, the exact opposite was true, which challenges the proposed link between perception and anatomy as a single visual anisotropy alone, such as the suggested perceptual compression along the horizontal meridian relative to the vertical meridian (or a smaller size of RFs or larger cortical magnification for the horizontal than for the vertical meridian), cannot explain these results.

## Method

### Participants

Twenty-two participants participated in Exp.1 (*M*_*age*_ = 28, *SD* = 12, seven males) and other twenty-two participants participated in Exp.2 (*M*_*age*_ = 28, *SD* = 9, six males). This sample size ensured a power of 0.80 (*α* = 0.05) for effect sizes of about *dz* = 0.55 for each experiment. As it was not possible to extract an expected effect size from previous research, assuming a medium effect size appeared a reasonable approach. All participants were recruited through the participant-acquisition system (SONA systems) of the University of Würzburg and received monetary compensation (5 Euro) for their participation. Informed consent was obtained from all subjects and/or their legal guardian(s). The study has been approved by the local ethics committee (Ethikkommission des Institutes für Psychologie der Humanwissenschaftlichen Fakultät der Julius-Maximilians-Universität Würzburg, GZEK 2020-88). All methods were performed in accordance with the relevant guidelines and regulations.

### Apparatus

Exp.1 was an online-experiment in which participants performed the experiment on their own computers. The spatial resolution of the most screens was 1920 × 1080 pixels (16 participants). The remaining screens had the resolutions of 1920 × 1200 (2 participants), 1600 × 900 (2 participants), 2256 × 1504 (one participant) and 1368 × 768 (one participant). Except for one screen (75 Hz), the refresh rate was about 60 Hz. Exp.2 was performed in a normally illuminated laboratory. In Exp.2, participants sat in front of a 23-inch monitor (Eizo, EV2303W; 1920 × 1080 pixels (px); 1 px = 0.2655 mm) at a distance of 56 cm. Participants’ head was supported by a chin rest. Program files were written using E-Prime software (Version 3.0; Psychology Software Tools, Pittsburgh, PA).

### Stimuli

All stimuli were presented on a gray background (with RGB color space coordinates 128, 128, 128). The fixation cross (7 × 7 px) and the number-sign symbols (18 px in height) were light gray, the question mark (22 px) was displayed in green. These stimuli were presented in the center of the screen. The main stimuli were Gabor patches with raised-cosine envelops (hemming) of 162 × 162 px in size (4.4° in Exp.2). The gratings were black-and-white on a gray background (with RGB coordinates 128, 128, 128) and were oriented either horizontally (90°) or vertically (0°). In each trial, one of the two Gabor patches (standard stimulus) had always a constant SF of 0.10 cycles per pixel (3.7 cycles per degree of visual angle in Exp.2). The SF of the other patch (test stimulus) varied between 0.05 and 0.15 cycles/px (1.8 and 5.5 cycles/degree in Exp.2) in steps of 0.01 (0.368 cycles/degree in Exp.2). The standard stimulus could appear either left or right, or above or below the fixation cross (counterbalanced for both dimensions). If the standard stimulus was a grating with vertical (horizontal) orientation, then the test stimulus was a grating with horizontal (vertical) orientation. The gratings appeared at a distance of 250 px (6.8° in Exp.2) from the fixation cross.

### Procedure and task

**Figure 1:**
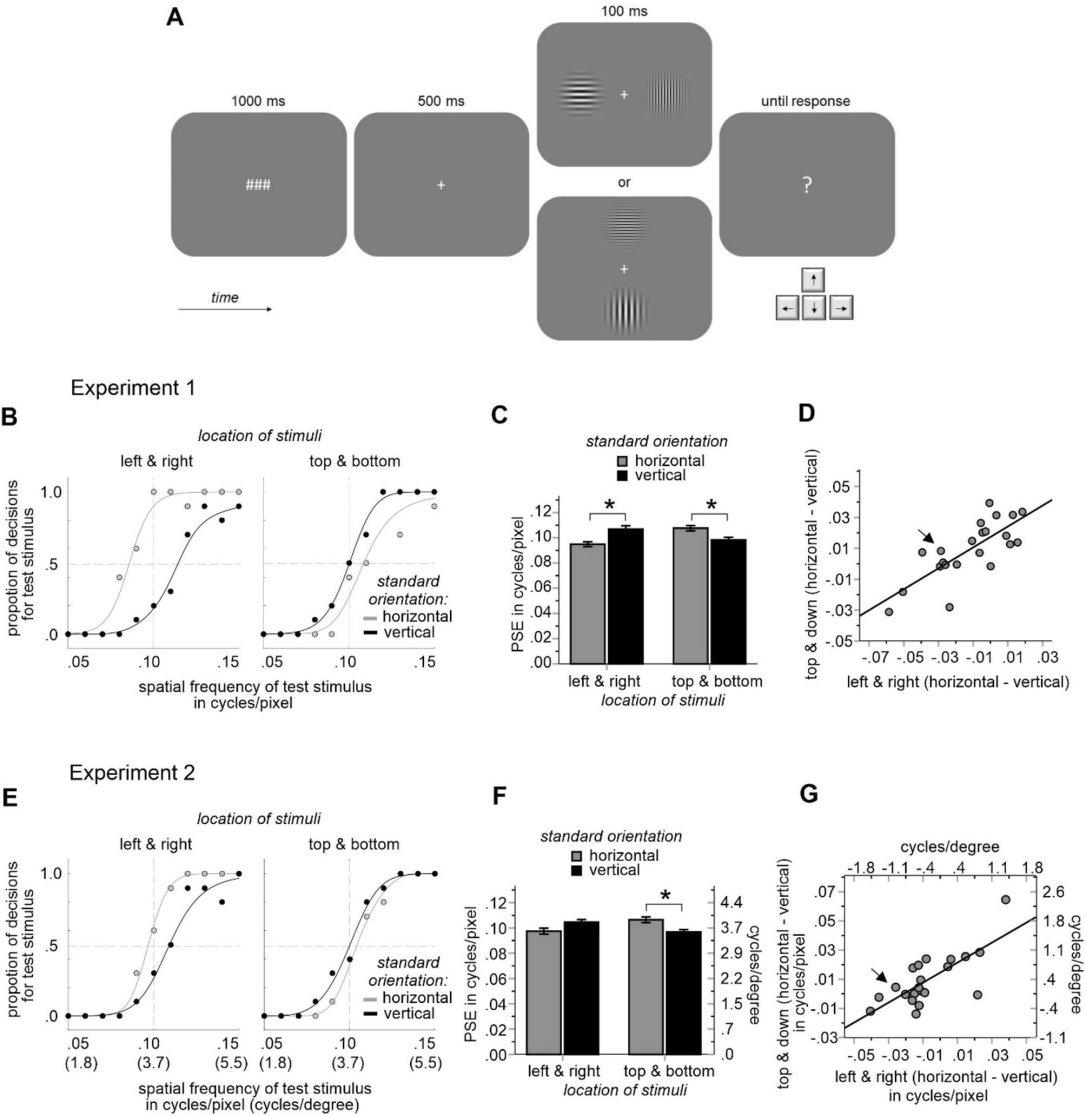
Procedure and results of the study. Main trial events (**A**) and results of Exp.1 (**B**–**D**) that was conducted online, and of Exp.2 (**E**–**G**) that was performed in a lab. Participants judged which of two Gabor patches presented either left and right of the fixation cross, or above and below it, is of a higher spatial frequency. (**B**,**E**) Shows the judgment data of a single participant for each experiment and corresponding psychometric functions. (**C**,**F**) Illustrates mean PSE values of all participants derived from psychometric functions fitted to the individual judgment data. (**D**,**G**) Shows individual PSE differences between horizontal and vertical orientations of the standard stimulus under both stimulus location conditions. Arrows denote the participants whose data are shown in (**B**,**E**). Error bars are standard errors. Asterisks denote statistical significance (p < 0.05). Stimuli shown in (**A**) are not drawn to scale.

Three number-sign symbols were initially displayed for 1 s. This arbitrary chosen symbol combination indicated a new trial (as in our previous studies e.g.^15^). Following an interval of 500 ms in which a fixation cross was visible, a pair of Gabor patches was flashed for 100 ms (while fixation cross remained visible). The Gabor patches were presented either left and right of the fixation cross or above and below it (random order). Finally, a question mark appeared and the participants had to indicate the location of the Gabor patch with a higher SF (see Fig. 1A). They were told to indicate the stimulus with a higher density of lines (i.e., that with thinner and more lines). The judgment was made by pressing arrow keys of the keyboard (left arrow key for left stimulus, right arrow key for right stimulus, up arrow key for the upper stimulus, and down arrow key for the lower stimulus). If participants pressed a wrong key (e.g., when gratings appeared left and right of the fixation cross and the upper or lower arrow key was pressed), error feedback was provided and the trial was repeated.

### Design

Overall, there were 44 experimental conditions: 2 location dimensions × 2 stimulus orientations × 11 levels of test stimulus. Each of these conditions was repeated 10 times (i.e., there were 10 trials per condition) and was presented in a random order. The main experiment had four blocks of trials including 110 trials each. Participants were asked to take breaks after each block. Before the main experiment started, participants performed 20 practice trials, in which we provided visual feedback about whether judgment was correct or not. These trials were not included in the analyses. During the main experiment, no feedback was given. Participants were asked to not to move their eyes and to always look at the fixation cross. In the online experiment (Exp.1), we also asked the participants to close all open applications and to ensure that they are not disturbed by other persons or their mobile phone. No specific instructions relating to viewing distance or lighting conditions were given.

### Data analysis

For each level of test stimulus, we computed the proportion of trials in which the test stimulus was judged as having a higher SF. This was done for each location and orientation condition. A local model-free fitting procedure^16^ was then used to estimate psychometric functions and to determine the points of subjective equality (PSE). The data of one of the participants of Exp.2 had to be excluded from analyses due to low discrimination performance (mean *r*^2^ below 3SD of the mean of all participants). The mean *r*^*2*^ of the remaining data amounted to 0.95 (*SD* = 0.04) in Exp.1 and 0.97 (*SD* = 0.02) in Exp.2. PSE values were analyzed using analyses of variance (ANOVA) and t-tests (two-tailed, reported p-values are not corrected for multiple comparisons).

## Results

Consistent with previous reports^12–14^, we observed that horizontal gratings were judged as having a lower SF than vertical gratings (of the same frequency). However, this was the case only when the stimuli were presented along the horizontal meridian. For stimuli presented along the vertical, horizontal gratings were judged as having a higher SF than vertical gratings. This relationship was expressed in a significant interaction between both critical experimental factors (i.e. orientation of the standard stimulus and location of stimuli) in a within subjects ANOVA of PSEs in both experiments, *F*(1, 21) = 50.91, *p* < 0.001, *η*_*p*_^*2*^ = 0.708 and *F*(1, 20) = 32.14, *p* < 0.001, *η*_*p*_^*2*^ = 0.616 (see Fig. 1B,C,E,F and Fig. S1 in the supplementary materials). For Exp.1, the ANOVA also revealed a significant main effect of location, *F*(1, 21) = 5.99, *p* = 0.023, *η*_*p*_^*2*^ = 0.222, indicating slightly larger PSEs when stimuli appeared above and below the fixation cross (vs. to the left and right of it).

In Exp.1, pairwise comparisons revealed significant differences between horizontal and vertical orientations of the standard stimulus for stimuli presented along the horizontal meridian, *t*(21) = 2.61, *p* = 0.016, as well as for stimuli presented along the vertical meridian, *t*(21) = 2.31, *p* = 0.031. In Exp.2, only the difference for the vertical meridian was significant, *t*(20) = 2.45, *p* = 0.024, but the difference for the horizontal meridian was not, *t*(20) = 1.72, *p* = 0.100.

Moreover, the judgment differences between horizontal and vertical orientations of the standard stimulus observed under both stimulus location conditions correlated highly with each other across participants in both experiments, *r* = 0.767, *p* < 0.001 and *r* = 0.735, *p* < 0.001 (see Fig. 1D,G). Participants who perceived horizontal gratings as coarser in one stimulus location condition tended to show the same effect in another stimulus location condition. This result indicates a consistency of individual judgments irrespective of whether the stimuli appeared along the horizontal or vertical meridian. Note that this outcome does not contradict the analyses of the mean values described above (as mean values reflect the intercept of a linear relation rather than its slope).

## Discussion

Visual performance varies across the visual field. It usually decreases with retinal eccentricity and is better along the horizontal than along the vertical meridian. These performance asymmetries are explained, in essence, by changes of spatial resolution of the visual system being expressed, e.g., in larger RF size and smaller cortical magnification for the visual periphery and along the vertical meridian^1–8^. Such visual field asymmetries imply not only differences between distant locations but also perceptual and neuroanatomical differences for the neighboring locations of the *same* area of the visual field. The visual processing of an object such as a square presented in the center of visual field, e.g., should go along with higher spatial resolution along its width than along its height (due to horizontal-vertical asymmetry). The present results, we believe, capture such an asymmetrical distribution of spatial resolution along the horizontal and vertical directions of an object in the peripheral regions of the visual field. In other words, they suggest *local* distortions of cortical maps in the visual periphery.

**Figure 2:**
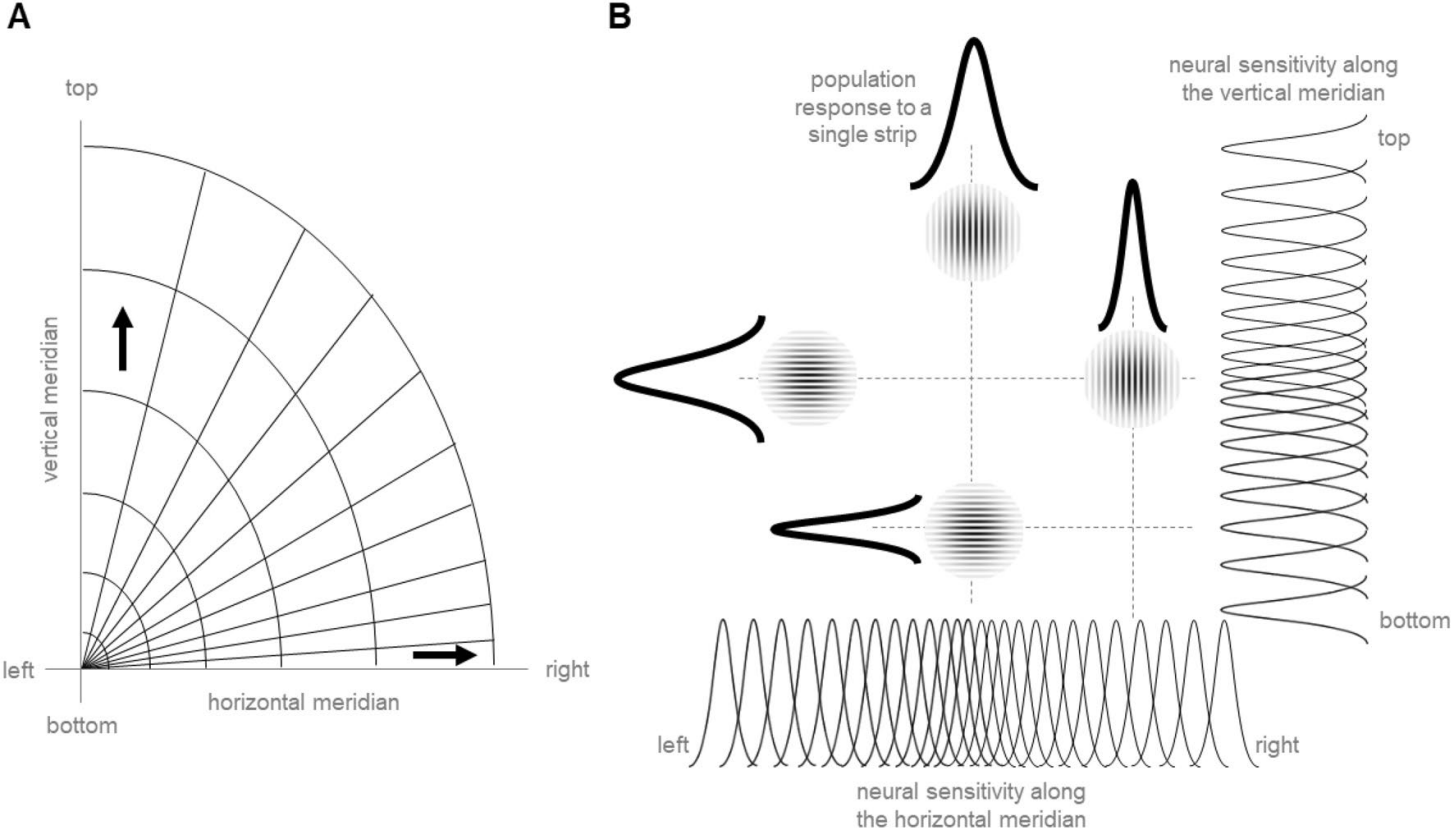
An explanation for why apparent spatial frequency varies depending on orientations and locations of two gratings. (**A**) Schematically illustrates an overall decrease in spatial resolution with eccentricity and a higher spatial resolution along the horizontal meridian in the right upper quadrant of the visual field (indicated by the density of adjacent lines). The map entails an asymmetry between the resolutions along the vertical and the horizontal axes (indicated by the arrows) that decreases for intercardinal meridians. (**B**) Shows how such a local asymmetry can be expressed in the perception of SF. Gray bell-shaped curves denote tuning curves (i.e., RF) of hypothetical neurons coding neighboring retinal locations. Black bell-shaped curves indicate population responses to a single strip of a grating. A higher spatial resolution along a certain dimension (i.e., smaller RF, larger density of RF, larger cortical magnification) produces a perceptual expansion along this dimension due to stronger population responses to single strips of the gratings. Accordingly, when the spatial resolution is lower (higher) along the horizontal than along the vertical axis for a certain location an object at this location is perceived to have a lower (higher) SF along the vertical than along the horizontal.

Such local horizontal-vertical differences are usually not considered when visual performance or neuroanatomy are compared across different spatial locations. Accordingly, a generally better performance along the horizontal than the vertical meridian, e.g., does not necessarily imply a better spatial resolution of the horizontal than of the vertical dimension for an object presented somewhere in the visual field. As illustrated in Fig. 2A, a roughly circular shape of the visual field rather suggests that the spatial resolution along the cardinal directions in a cortical map can be lower than in the opposite directions. Such a local asymmetry can explain the present results. Vertical gratings are perceived to have a lower SF (than horizontal gratings) when they appear along the vertical meridian because the spatial resolution at stimulus location is higher along the horizontal than along the vertical and vice versa. Figure 2B illustrates the corresponding basic model.

It is important to note that the present results demonstrate an effect in perceived SF. Accordingly, the suggested explanation may be limited to visual appearance and not necessarily generalize to visual performance. Yet, we believe that our conclusions may also hold for visual performance. Consider, e.g., that performance differences across the visual field are reported for different orientations and tilt angles of stimuli^17–21^. This fact is taken into account in Fig. 2A by an overall decrease in spatial resolution with eccentricity and an overall higher spatial resolution at horizontal than at vertical meridian (cf. the extents of areas surrounded by lines either along each meridian or between them). To better visualize a horizontal meridian advantage in visual discrimination of a vertically oriented stimulus, e.g., just mentally shift the x-coordinates of the circular lines to the left so that the spatial distances between them become even smaller along the horizontal than the vertical meridian but the local asymmetry (indicated by the arrows in Fig. 2A) remain preserved. The more important prediction here is that in addition to this general advantage of the horizontal meridian, vertically oriented stimuli should be more easily discriminated along the vertical meridian than horizontally oriented stimuli and vice versa due to local asymmetries in spatial resolution.

In fact, the present results resemble the so-called radial bias—enhanced perceptual sensitivity to radially-oriented as compared to tangentially-oriented contours (i.e. to stimuli oriented collinear as compared to orthogonal to a line intersecting the fixation point)^22–24^. Horizontally (vertically) oriented gratings can be construed as oriented radially (tangentially) when presented along the horizontal meridian. The opposite is true when the same gratings are presented along vertical meridian. Considered from this perspective, the present results revealed that gratings oriented radially were perceived as having a lower SF than gratings oriented tangentially. This outcome is consistent with the radial bias if a higher spatial resolution entails a lower apparent SF as we suggest (see Fig. 2). In other words, our explanation delineated in Fig. 2 can also be applied to and explain the radial bias. Accordingly, enhanced sensitivity to radially-oriented contours at a certain visual location is basically due to higher spatial resolution along the tangential direction at this location (i.e. due to local cortical distortions). As a result, deviations of the stimulus orientation from the radial orientation, e.g., can better be detected than deviations from the tangential orientation^24^. This reasoning points to an interesting question for future studies, namely whether spatial frequency is perceived as coarser along the radial direction when stimuli are presented beyond the cardinal meridians.

Our approach is consistent with earlier observations of lower apparent SF for horizontal than for vertical gratings presented along the horizontal meridian^13,14^. The proposed link between (low) spatial resolution and (small) apparent size of objects can explain why apparent SF increases with eccentricity^12^, and is in line with the tendency to perceive stimuli as smaller in peripheral compared to central vision as well as along the vertical compared to horizontal meridian^25,26^. Because the overall spatial resolution gradually decreases from the horizontal to the vertical meridian (i.e. along the arcs, see Fig. 2A) our approach also indicates why perceptual and anatomical asymmetries hold especially for cardinal axes of the visual field and decrease gradually with deviations from these axes^6^. This property predicts a gradual drop in the extent of the horizontal-vertical anisotropy at intercardinal meridians, in principle, for any stimulus orientation, including tilt angles of 45° from the horizontal e.g.^18^ (cf. also the last but one §). But note here than beyond changes in overall spatial resolution local asymmetries should affect visual performance consistent with the radial bias mentioned in the previous paragraph.

Interestingly, it has been reported that apparent SF is higher for gratings presented along the horizontal than along the vertical meridian^27^. In that study, however, only vertically oriented gratings were used. If we only consider vertically oriented gratings in our experiments (and ignore the horizontally oriented gratings as if they served as a reference stimulus), then our results approximate the results of that study. As shown in Fig. 1 (black bars in C and F), the PSE for the vertically oriented gratings is higher for the horizontal than for the vertical meridian suggesting that perceived SF of vertical gratings was higher at the horizontal than at the vertical meridian. Moreover, the explanation we suggest for our results also applies to the main results of the mentioned study. The perceived SF of vertical gratings is higher at the horizontal meridian because the spatial resolution is higher along the vertical than along the horizontal axis of the stimuli, while the opposite is true for the vertical meridian (higher horizontal resolution; see also population responses to the vertical gratings in Fig. 2B).

It should be noted that in Exp.2 the PSE difference between the orientation conditions for stimuli presented along the horizontal meridian did not reach the significance threshold (when a two-tailed test was applied). Thus, one part of the results of the online-experiment (Exp.1) was not replicated in the lab (Exp.2). This could question the reliability of the findings. This particular aspect of the data (i.e. judgments of the horizontal gratings as coarser when presented along the horizontal meridian) has already been reported previously^12–14^ (see also “Introduction”). Thus, the effect seems reliable, but its size was obviously underestimated in Exp.2. One possible reason for this can be seen in Fig. 1D,G—there was a substantial number of participants who showed an opposite pattern of results as compared to the results of the means. This individual variability may also limit the generalizability of the main findings. Interestingly, participants were rather consistent in their judgment behavior regardless of the grating locations (see correlation analyses and Fig. 1D,G) which points to interindividual differences in the brain anatomy^8,25,28^. For example, in participants who tended to perceive horizontal gratings as coarser under both location conditions the local spatial resolution could generally be higher along the vertical than along the horizontal (this can be imagined by mentally removing an oblique line next to the vertical line in Fig. 2A that would inverse the asymmetry between the horizontal and vertical resolutions indicated by the vertical arrow).

Another possible issue that can be raised is related to potential eye movements, which might systematically affect the results. This possibility is rather unlikely due to the timing and the specific design of the experiments that aimed to avoid such influences. For example, the stimuli were presented pairwise and for a duration that is below the usual reaction time of eye-movements. Moreover, to solve the task the participants had to attend to both stimuli and it was unpredictable whether the stimuli will appear along the horizontal or vertical meridian and which of them will be horizontally or vertically oriented.

Overall, the main limitation of the current study is that our approach rests on an assumed link between psychophysical effects and their neurophysiological basis that is not fully understood. Accordingly, the suggested explanations have to be considered as preliminary and appropriate to the extent this link is justified.

### Supplementary Information

Supplementary Figure S1.

## Data Availability

The data and program scripts for both experiments are publicly available via the Open Science Framework (https://osf.io/qegdu/). The study was not preregistered.

## References

[1] Strasburger H, Rentschler I, Jüttner M (2011). Peripheral vision and pattern recognition: A review. J. Vis..

[2] Himmelberg MM, Winawer J, Carrasco M (2023). Polar angle asymmetries in visual perception and neural architecture. Trends Neurosci..

[3] Duncan RO, Boynton GM (2003). Cortical magnification within human primary visual cortex correlates with acuity thresholds. Neuron.

[4] Harvey BM, Dumoulin SO (2011). The relationship between cortical magnification factor and population receptive field size in human visual cortex: Constancies in cortical architecture. J. Neurosci..

[5] Himmelberg MM, Kurzawski JW, Benson NC, Pelli DG, Carrasco M, Winawer J (2021). Cross-dataset reproducibility of human retinotopic maps. NeuroImage.

[6] Benson NC, Kupers ER, Barbot A, Carrasco M, Winawer J (2021). Cortical magnification in human visual cortex parallels task performance around the visual field. eLife.

[7] Silva MF, Brascamp JW, Ferreira S, Castelo-Branco M, Dumoulin SO, Harvey BM (2018). Radial asymmetries in population receptive field size and cortical magnification factor in early visual cortex. NeuroImage.

[8] Himmelberg MM, Winawer J, Carrasco M (2022). Linking individual differences in human primary visual cortex to contrast sensitivity around the visual field. Nat. Commun..

[9] Blakemore C, Sutton P (1969). Size adaptation: A new aftereffect. Science.

[10] Virsu V (1974). Letter: Dark adaptation shifts apparent spatial frequency. Vis. Res..

[11] Kulikowski JJ (1975). Apparent fineness of briefly presented gratings: Balance between movement and pattern channels. Vis. Res..

[12] Georgeson MA (1980). Spatial frequency analysis in early visual processing. Philos. Trans. R. Soc. Lond. Ser. B.

[13] Bowker DO (1981). Variations in apparent spatial frequency with stimulus orientation: I. Incidence of the effect in the general population. Percept. Psychophys..

[14] Bowker DO (1981). Variations in apparent spatial frequency with stimulus orientation: II. Matching data collected under normal and interferometric viewing conditions. Percept. Psychophys..

[15] Kirsch W, Heitling B, Kunde W (2018). Changes in the size of attentional focus modulate the apparent object's size. Vis. Res..

[16] Zychaluk K, Foster DH (2009). Model-free estimation of the psychometric function. Atten. Percept. Psychophys..

[17] Baldwin AS, Meese TS, Baker DH (2012). The attenuation surface for contrast sensitivity has the form of a witch's hat within the central visual field. J. Vis..

[18] Barbot A, Xue S, Carrasco M (2021). Asymmetries in visual acuity around the visual field. J. Vis..

[19] Carrasco M, Talgar CP, Cameron EL (2001). Characterizing visual performance fields: Effects of transient covert attention, spatial frequency, eccentricity, task and set size. Spatial Vis..

[20] Corbett JE, Carrasco M (2011). Visual performance fields: Frames of reference. PLoS ONE.

[21] Purokayastha S, Roberts M, Carrasco M (2021). Voluntary attention improves performance similarly around the visual field. Atten. Percept. Psychophys..

[22] Bennett PJ, Banks MS (1991). The effects of contrast, spatial scale, and orientation on foveal and peripheral phase discrimination. Vis. Res..

[23] Sasaki Y, Rajimehr R, Kim BW, Ekstrom LB, Vanduffel W, Tootell RB (2006). The radial bias: A different slant on visual orientation sensitivity in human and nonhuman primates. Neuron.

[24] Westheimer G (2003). The distribution of preferred orientations in the peripheral visual field. Vis. Res..

[25] Moutsiana C, de Haas B, Papageorgiou A, van Dijk JA, Balraj A, Greenwood JA, Schwarzkopf DS (2016). Cortical idiosyncrasies predict the perception of object size. Nat. Commun..

[26] Schwarzkopf DS (2019). Size perception biases are temporally stable and vary consistently between visual field meridians. Perception.

[27] Montaser-Kouhsari L, Carrasco M (2009). Perceptual asymmetries are preserved in short-term memory tasks. Atten. Percept. Psychophys..

[28] Song C, Schwarzkopf DS, Rees G (2013). Variability in visual cortex size reflects tradeoff between local orientation sensitivity and global orientation modulation. Nat. Commun..

