# Supplementary material for: Human perception of spatial frequency varies with stimulus orientation and location in the visual field
Source: Sci Rep. 2023 Oct 17;13:17656. doi: 10.1038/s41598-023-44673-8 (PMC10582250; doi:10.1038/s41598-023-44673-8)
Supplement: Supplementary file 1 — Supplementary Figure S1. [file 41598_2023_44673_MOESM1_ESM.docx]

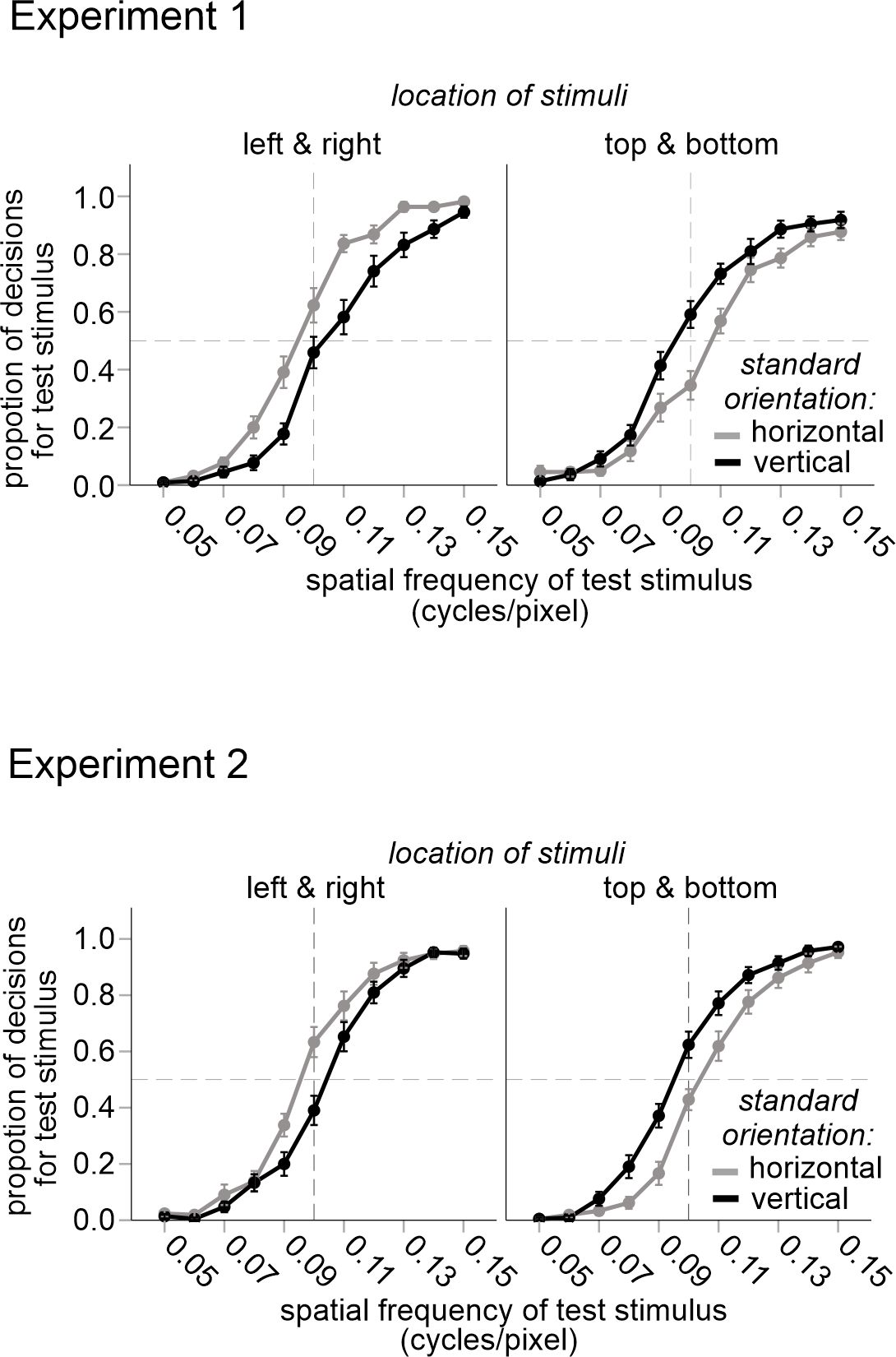


***Figure S1.*** *Shown are mean proportions of decisions fort the Gabor patch that served as a test stimulus as a function of the spatial frequency of the test stimulus, orientation of the standard stimulus (always orthogonally rotated in respect to the test stimulus) and the locations of both stimuli.*
